# Archaeological Remains Accounting for the Presence and Exploitation of the North Atlantic Right Whale *Eubalaena glacialis* on the Portuguese Coast (Peniche, West Iberia), 16th to 17th Century

**DOI:** 10.1371/journal.pone.0085971

**Published:** 2014-02-05

**Authors:** António Teixeira, Rui Venâncio, Cristina Brito

**Affiliations:** 1 Instituto da Conservação da Natureza e das Florestas, Ministério da Agricultura e do Mar, Lisboa, Portugal; 2 Museu Municipal de Peniche, Câmara Municipal de Peniche, Peniche, Portugal; 3 Centro de História de Além-Mar (CHAM), Faculdade de Ciências Sociais e Humanas, Universidade Nova de Lisboa and Universidade dos Açores, Lisboa, Portugal; University of Oxford, United Kingdom

## Abstract

The former occurrence of the North Atlantic right whale *Eubalaena glacialis* on the Portuguese coast may be inferred from the historical range of that species in Europe and in NW Africa. It is generally accepted that it was the main prey of coastal whaling in the Middle Ages and in the pre-modern period, but this assumption still needs firming up based on biological and archaeological evidence. We describe the skeletal remains of right whales excavated at Peniche in 2001–2002, in association with archaeological artefacts. The whale bones were covered by sandy sediments on the old seashore and they have been tentatively dated around the 16th to 17th centuries. This study contributes material evidence to the former occurrence of *E. glacialis* in Portugal (West Iberia). Some whale bones show unequivocal man-made scars. These are associated to wounds from instruments with a sharp-cutting blade. This evidence for past human interaction may suggest that whaling for that species was active at Peniche around the early 17th century.

## Introduction

In sharp contrast to its current status of endangered species [1] the North Atlantic right whale *Eubalaena glacialis* was a comparatively well-known cetacean in the marine fauna of Medieval Europe. It was the primary target of several old whaling settlements in the Bay of Biscay with capture techniques pioneered by the Basques around the 10th century [2], [3]. The whales were sighted from lookouts in vantage points ashore and because they are slow swimmers (9–11 km/h) they could be approached within spearing distance by the whalers in small rowing boats [4]–[6]. Combination of coastal habitat, large size and rotund bodies with huge reserves of subcutaneous fat made these whales economically valuable prey. The dead whales remained buoyant after being killed. They could be picked up by the whalers and processed at suitable places ashore. These were indeed the “right” whales to catch.

This was a migratory species in the temperate regions of the NE Atlantic and adjacent seas. There were seasonal movements between feeding areas, mostly in the northern latitudes (up to *ca*. 60°N) and calving grounds in the southern part of their range. Whaling statistics studied by Brown [7] show records around Ireland in early June and movements further north to Scotland. They were captured in Hebridean waters from late June through July [8] before heading south in the autumn. They were close to the shores of Cantabria (N. Spain) from November to May and they were exploited there by Old Basque whalers. Their range reached further south and study of logbooks from 18th and 19th century American or “Yankee” pelagic whaling suggests an important wintering ground in the Cintra Bay region, between 22°–24°N along the coast of north-western Africa. Right whales could be observed there from November to April, with a possible peak in sightings in January–February [9].

Late in the 19th century Graells [10] did claim that Old Basque whaling was sustainable. It had been going on for many years in the Bay of Biscay, until the local fishermen redirected their attention to new and very profitable long-distance fisheries overseas. He also stated that many whales were still present in the area when the activity was abandoned in the mid 17th century. Captures from Basque whaling in the Bay of Biscay have been associated to the collapse of the coastal whaling industry there [11], [2] but this view remains controversial and was not endorsed by Aguilar [5]. The numbers of right whales killed in the coastal waters of northern Iberia probably were quite low, and catches in the region had been going on for seven centuries before the activity finally came to an end. According to Aguilar [5] a few whales were still harvested around the nineteenth century. If these claims are true, the major cause for depletion of stocks and the final blow to this population may relate to unsustainable captures by pelagic whalers operating in the wintering quarters off the NW African coast [12], [13].

The main objective of our research is to expand knowledge about the former land-based whaling in Portugal and to investigate the biological aspects of the activity, which includes checking the target species. Ancient written records are an important source of information but access to the skeletal remains of old specimens is also necessary. We address the historical presence and relative abundance of whale species in Portugal, including the former occurrence of right whales in the medieval and early modern periods.

## Methods

**Figure 1:**
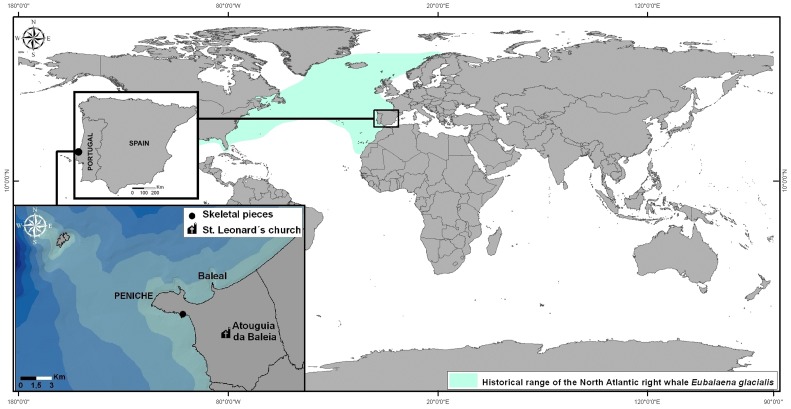
Map showing the excavation site in Peniche (Portugal, mainland) where the whale bones were found in 2001–2002. Also included the position of St. Leonard's church (Atouguia da Baleia) where a jawbone of a large Balaenopterid whale is displayed. The oceanic area painted in light green is the documented historical range of the North Atlantic right whale *Eubalaena glacialis*.

**Figure 2:**
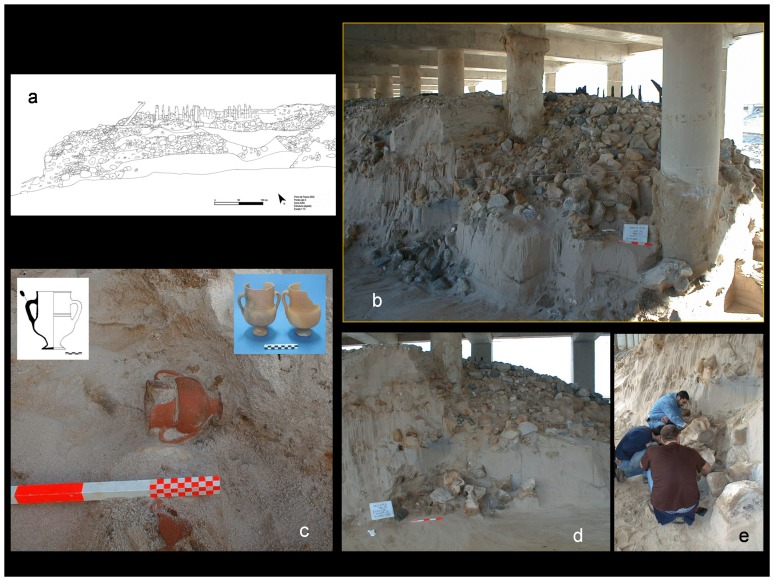
The excavation site at Peniche (W. Portugal) showing where the whale bones and archaeological materials (pottery and structures) were found in 2001–2002. (a) graphic representation of an old road structure (lateral view) showing fill-in with rubble materials and a line of wooden poles set up vertically in the soft sediment for support; (b) The concrete piers of the new harbour pushing through remains of an old road structure underneath; (c) *In situ* photograph of a well preserved ceramic mug from late medieval or pre-modern chronology, still imbedded in sediment (inserts show details of the type); (d) Sample of whale bones pilling up in sand, *ca.* 3 m bellow the top layer of the old road structure; (e) Members of the archaeological team excavating the worksite in Peniche in 19th April 2002 (Joaquim Meireles has given written informed consent, as outlined in the PLOS consent form, to publication of their photograph).

The buried remains of an old road structure in a coastal dune habitat were excavated in 2001–2002 at Peniche (Portugal, West Iberia). These archaeological finds came up in line with public works to expand the harbour facilities in this fishing village (Fig. 1). Whale bones and many fragments of household pottery were also found and have been collected at the worksite. They are now preserved in the local Museum (*Museu Municipal de Peniche*). This intervention was authorised according to national legislation and licenses from the Portuguese Institute of Archaeology (IPA, currently renamed IGESPAR) were issued in 16 April 2002. All the archaeological pieces were photographed at site, and the images obtained also included some team members (Joaquim Meireles has given written informed consent, as outlined in the PLOS consent form, to publication of their photograph). The pieces collected did receive an inventory number, with a common designation PP.02 followed by an individual sequence of three digits (443 to 568). Further skeletal remains of whales were identified at the worksite, trapped in concrete by the new structures under construction. They were photographed and left buried *in situ*. The pottery fragments were widely scattered around and some of them stood close to the skeletal remains of large whales and domestic ungulates (Fig. 2). Caution is called upon when attempting to extract chronological sequences from the relative positions of archaeological materials found in non-consolidated sediments. In this case, the deposition of sand was probably affected by tidal currents and influenced by storms, but the analysis of the materials found is very much worthwhile. Checking their occurrence against descriptions in old documents helps establishing an historical time frame. In the future, these bones may be re-examined in the light of more precise techniques (radiocarbon dating and eventually DNA fingerprinting).

Old administrative rules issued by the Crown (*forais*) are an important source of historical information. They must be checked for the presence (or absence) of former dispositions about whaling activities in a given area. We shall also keep in mind that the shape and position of the seashore around Peniche has changed dramatically in historical times. Sandy sediments have accumulated in some areas, pushing the contour of the shore seaward for distances of several hundred metres. This was checked in the historical sources and from reliable illustrations in old maps.

## Results

### Archaeological framework

Public works carried out in 2001–2002 to expand the fishing harbour facilities at Peniche did expose sections of an old road structure buried in sandy sediments. This structure had been installed with wooden piers pushing vertically through a dune habitat and presumably it was built to improve the circulation of people and cargo over stretches of soft sand. The building materials and techniques used in this construction suggest that it was installed possibly in the 16th–18th centuries [14]. Within close range and *ca*. 3 metres below the top layers of the structure there were also many fragments of pottery household buried in sand. Most of these artefacts could be dated to late medieval or early modern manufacture, but there were also materials from other chronologies. Such a concentration of so many pottery fragments in a comparatively small area on the old beachfront may suggest a shipwreck context. This is empathized by almost no traces of marine fauna on the pottery items recovered, suggesting their quick inclusion in the sediment. This would be explained by the simultaneous loss of cargo transported in one or more ships using the seaport at Peniche, or trying to make a safe passage to the ancient port of Atouguia upstream. Skeletal remains of large whales and a few bones of domestic ungulates were also excavated in this area. Some bones have been found close to pottery fragments dated to a period that spans from the mid 15th century to the early decades of the 16th century. The whale bones may have been discarded on the beach. If this was the case, possibly they were later re-utilized to consolidate the foundations of the old road when it was built in the dune system [14]. The archaeological context of these bones is consistent to dating them around the 15th to 17th centuries.

**Figure 3:**
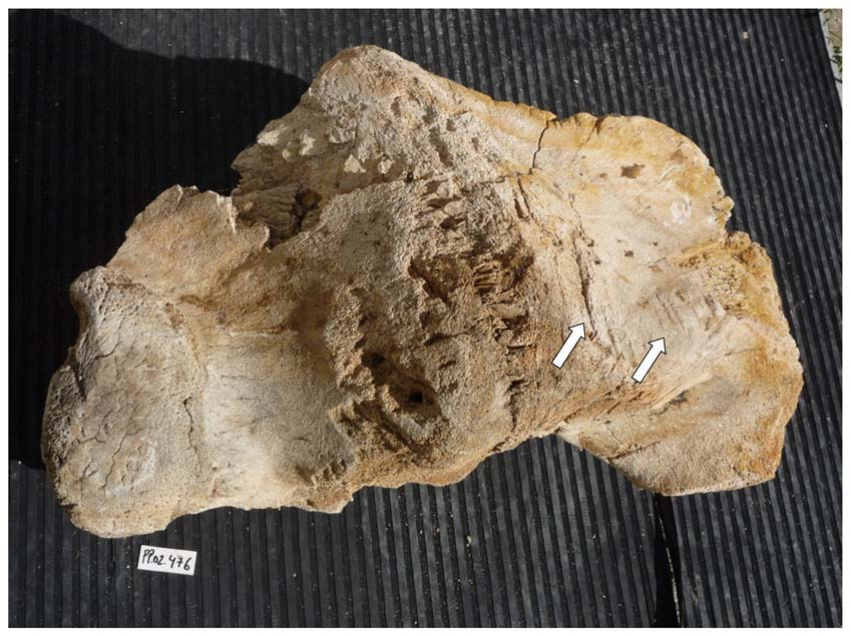
PP.02.476 is part of the occipital bone of a large right whale *Eubalaena glacialis* excavated at Peniche in 2001–2002 (right condyle to the left in this picture). Scars in the bone are visible in the foreground (white arrows) and probably they were caused by axe blows or flensing marks. For scale, the small white tablet in the bottom left (with characters PP.02.476) is 5.0×2.2 cm.

**Figure 4:**
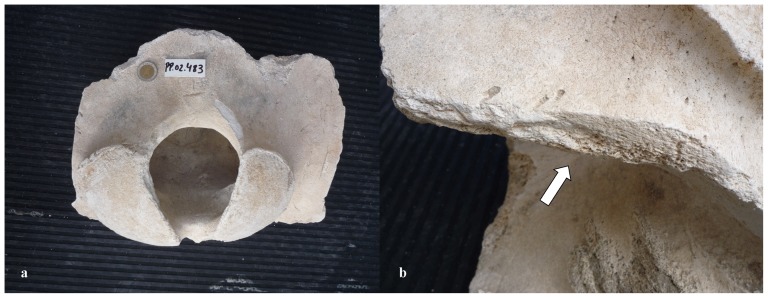
PP.02.483 is part of the occipital of a right whale *Eubalaena glacialis* excavated at Peniche in 2001–2002. (a) posterior view shows *foramen magnum* and two condyles; (b) close-up showing straight (man-made) cuts through the bone (arrow). The 2 euro coin (diameter 2.58 cm) was included for scale in photo a.

**Figure 5:**
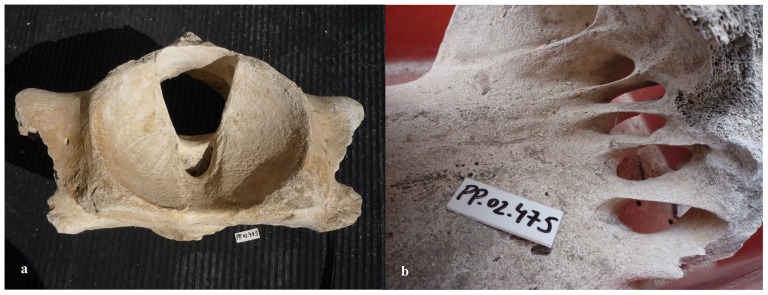
PP.02.475 is the neck of a right whale *Eubalaena glacialis* excavated at Peniche in 2001–2002. (a) the Atlas in frontal view shows two concave condyle-bearing surfaces; (b) close-up from the rear, detailing the dorsal surfaces and right-hand processes of the atlas fused to other (2–7) cervical vertebrae. The small white tablet in the photos (with characters PP.02.475) is 5.0×2.1 cm.

**Figure 6:**
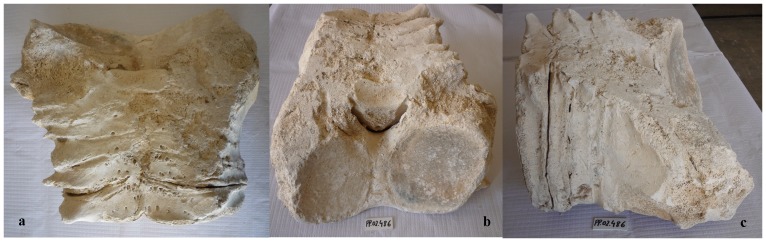
PP.02.486 is the neck of another North Atlantic right whale *Eubalaena glacialis* excavated at Peniche in 2001–2002. Despite intense weathering this photo shows the ankylosis (fusion) reaching through the whole group of (seven) cervical vertebrae: (a) the neck vertebrae in dorsal view; (b) remnants of the atlas and condyle bearing surfaces in frontal view; (c) lateral view. The small white tablet included for scale in photos b and c (with characters PP.02.486) is 5.0×2.2 cm.

**Figure 7:**
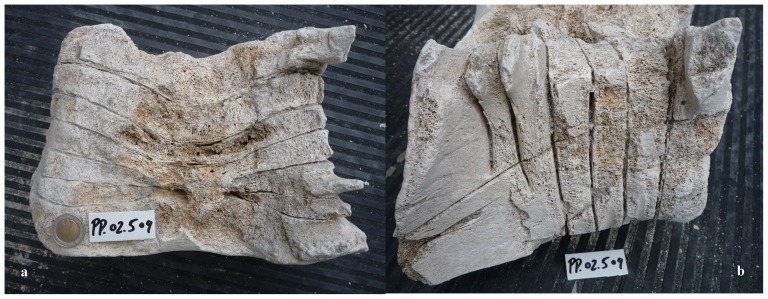
PP.02.509 was excavated at Peniche in 2001–2002 and shows the fused neck vertebrae of a third specimen of the North Atlantic right whale *Eubalaena glacialis*. Despite intense weathering the ankylosis (fusion) of all seven cervical vertebrae remains visible and is characteristic of adult right whales: (a) ventral side; (b) lateral view. The 2 euro coin (2.58 cm diameter) was included for scale in photo a. The small white tablet (with characters PP.02.509) in the two photos is 5.0×2.2 cm.

**Figure 8:**
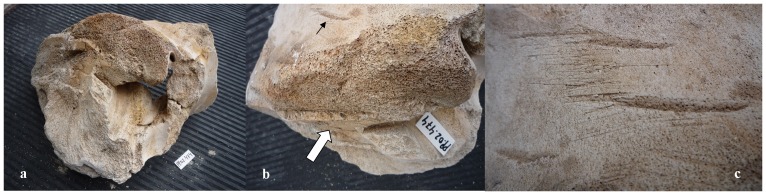
PP.02.474 is one fragment of a whale skull excavated at Peniche in 2001–2002. (a) compact bone structure with hollow centre; (b) detail showing man-made incisions in the bone with straight cuts visible in the foreground (white arrow); they are quite different from the deep incisions seen cutting through the bone surface (black arrow); (c) close-up showing large deep cuts (axe or flensing tool) alongside shallow and much thinner cuts, possibly made with a small hand-held knife. The small white tablet with characters PP.02.474 (included in photos a and b) is 5.0×2.2 cm.

Our sample of bones collected at the worksite includes a total of 91 pieces (Table 1). There are 80 bone fragments of large cetaceans and 11 of large terrestrial vertebrates (presumably domestic ungulates). At least three different whale specimens are involved. Parts PP.02.476 (Fig. 3) and PP.02.483 (Fig. 4) were in the occipital region of distinct whale specimens. One of these (PP.02.483) shows the two condyles and the spinal *foramen magnum*. Our sample also includes three sets of the cervical vertebrae of large whale specimens. Parts PP.02.475 (Fig. 5), PP.02.486 (Fig. 6) and PP.02.509 (Fig. 7), are a good fit to the occipital condyles already described. Parts PP.02.483 and PP.02.486 were found in close proximity and they have an exceptionally good match in shape and size. Some bones in our sample have a wealth of linear incisions on the bone surface. These cuts are obviously man-made. Parts such as PP.02.474 (Fig. 8) and many other show this striking feature. There are wide cuts reaching deep into the bone and they were probably made with an axe-like instrument or large flensing tool. Many shallow and thinner cuts are also visible on some bone surfaces and possibly these were made with a sharp hand-held knife.

**Table 1:** A list of the bones excavated at Peniche in 2001–2002.

Bone categories	No. of specimens
**Whale bones**	**80**
Skull bones	
Occipital region	4
Other	10
Neck vertebrae (fused cervicals - 1st through to the 7th)	3 sets
Ulna	1
Large vertebrae (fragments)	11
Vertebral disc (growth plate)	1
Rib (fragments)	8
Other (small bone fragments)	42
**Bones of terrestrial vertebrates**	
Domestic ungulates	**11**
**Total**	**91**

They are now preserved in the collections of the local Museum (MMP).

**Figure 9:**
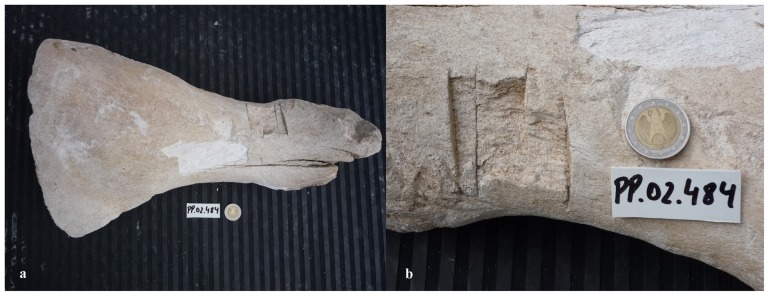
PP.02.484 is the ulna from the left forearm of a large North Atlantic right whale *Eubalaena glacialis* excavated at Peniche in 2001–2002. (a) there is a concentration of straight man-made cuts on the leading edge of this bone (carpal joint to the left and posterior edge pointing downwards in this photo); (b) close-up to show sharp, man-made cuts in the anterior edge of the ulna (facing downwards in this photo). The lighter coloured patch visible in the two photos denotes a recent (2013) loss of material from the surface layers of this bone. The 2 euro coin has a diameter of 2.58 cm and was included in the photos for scale.

**Figure 10:**
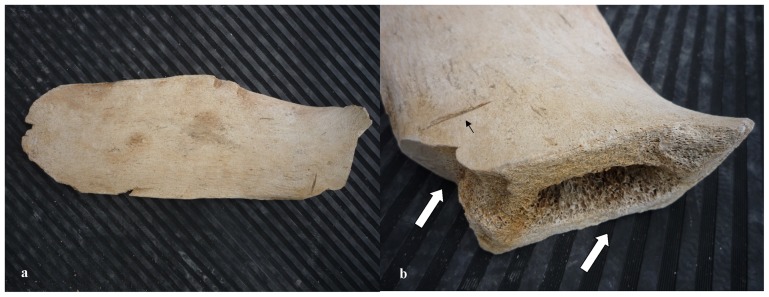
PP.02.503 is: (a) spinous process from one vertebra of a large whale excavated at Peniche in 2001–2002; (b) detail shows strait edges and clean cut sections (white arrows); this bone was deliberately cut off from the main body in the vertebra; a linear incision scratches the bone surface (small black arrow). This bone is 44 cm long and 16 cm wide.

PP.02.484 (Fig. 9) is the ulna from the left forelimb of a large whale. Measurements are summarized in Table 2. Our sample includes eleven fragments of large whale vertebrae. PP.02.493 is a well-preserved vertebral disc (growth plate). It is 2 cm thick and its contour line has an elliptical shape (31×23 cm). Eight bones in the sample are rib fragments. They certainly were in the skeleton of one or more whales with large body size. PP.02.496 is 57.5 cm long and has an oval cross-section (6.5×4.5 cm). This is the longest piece in the collection. PP.02.503 (Fig. 10) is the spinous process of one vertebra that was in the skeleton of a large whale. This bone has been deliberately cut off as illustrated by sharp (man-made) scars with straight edges. The same type of scars is also present in part PP.02.489 and in many other. The sample includes forty two bone fragments of comparatively small size. They were in the skeleton of large whale specimens but their anatomical position was not investigated in detail.

**Table 2:** Measurements of selected whale bones excavated at Peniche in 2001–2002.

Specimen ref. MMP	Skeletal part	Length (cm)	Width (cm)	Height (cm)	Notes
**PP.02.474**	**skull fragment**	42	36	29	has many cut marks
**PP.02.475**	**cervical vertebrae** (anastomosed)	34	69	45	matches PP.02.476
	cup of condyles (2×)	-	34	17	
**PP.02.476**	**occipital bone** (posterior area)	40 +	70 +	-	matches PP.02.475
	condyle (right condyle only)	23	13	-	
	foramen magnum	-	-	-	
	condyles+foramen magnum	-	-	-	
**PP.02.483**	**occipital bone** (posterior area)	33 +	37 +	-	matches PP.02.486
	condyles	18	12	-	
	foramen magnum	-	12	10.5	
	condyles+foramen magnum	21	29	-	
**PP.02.484**	**ulna** (left forearm)	47	28	-	
**PP.02.486**	**cervical vertebrae** (anastomosed)	33	42 +	-	matches PP.02.483
	cup of condyles (2×)	21	15	-	
**PP.02.493**	**vertebral growth plate** (unfused)	31	23	2	skeleton still growing
**PP.02.503**	**vertebra ‘wing’** (spinous process)	44	16	**-**	11,5×7 cm at base
**PP.02.509**	**cervical vertebrae** (anastomosed)	20	32	-	-

They are preserved in the collections of the local Museum (MMP). Overall length of the anastomosed (fused) neck vertebrae was measured on the longitudinal axis. Width was measured at the anterior end of the condyle articulation region in the atlas.

### Identification of the species

There are no complete skulls available for examination and only a few shattered fragments were found. Jawbones (or identifiable fragments) are missing in the sample and no teeth of cetaceans could be found anywhere around the worksite. No vestiges of baleen plates were found either. Many bone fragments are comparatively small and uncharacteristic, but all of them show typical cetacean structure and morphology. They are evidence that whale specimens with large body size (10 metres or longer) were present but are little help to the identification of the species involved, unless we go beyond the old classic techniques in comparative anatomy. However, we have three blocks of ankylosed cervical vertebrae (PP.02.475; PP.02.486; and PP.02.509) and these may be used in the identification of the whale species. As a starting point, it should be noted that all cetacean bones in the sample have a spongy structure that is typical in this group of marine mammals. The bones are comparatively light in weight and not very consistent, being quite susceptible to structural damage from undue manipulation. They show signs of intense weathering and there is no trace of any cetacean oil retained in the bone structure. We stand far from the petrified bones of ancient (extinct) cetaceans. Therefore we must resume our exercise in identification only to the extant species. Some bones have linear incisions that are obviously man-made. This demonstrates interaction with modern man equipped with sharp iron blades. All these conditions combine to set up a possible time frame of only a few centuries BP.

The whale specimens in our sample were obviously large and this stands out clearly from measurements of their bones in Table 2. Based on size criteria alone, we may exclude all modern cetacean species unable to reach body length in excess of 10 metres. Therefore we have a choice limited only to a few possible species. The initial short list includes sperm whale and the larger mysticetes. Ankylosis of the cervical vertebrae excludes the rorquals. The Balaenopteridae are slim-shaped cetaceans with a comparatively long neck and they have separate (unfused) cervical vertebrae. Their skeleton retains these characteristics for life. Further to descriptions in the classic literature [15] this point was checked through comparative study of specimens in museum collections. These include one large (20 m) adult fin whale *Balaenoptera physalus* in the Zoological Museum of the Coimbra University, and a smaller (13,80 m) specimen of the same species in the Zoological Museum of the Porto University. The gray whale *Eschrichtius robustus* was considered initially because they had a North Atlantic population until the 17th or 18th centuries [3]. However, gray whales must be excluded from our exercise because they have an elongated neck and free (non-ankylosed) cervical vertebrae like the Balaenopteridae [16]. The sperm whale *Physeter macrocephalus* occurs in the area but its cervical vertebrae are only partly fused; the atlas is separate from the rest, while the 2nd through to the 7th are usually fused together. Flower [15] has pointed out that “the Cachalot, or Sperm Whale (*Physeter*) presents a condition not met with in any other known cetacean: the atlas is free, and all the other neck vertebrae are completely united”. This condition is not compatible to parts PP.02.475, PP.02.486, and PP.02.509 in our sample (Figs. 5, 6 and 7). All of them show full ankylosis in the whole set of seven cervical vertebrae. As a consequence, sperm whale must be rejected.

From anatomical evidence we may therefore conclude that the whale bones excavated at Peniche in 2001–2002 are of right whales (Balaenidae). There are two candidate species in this family. The bowhead whale *Balaena mysticetus* has a circumpolar distribution and its range is restricted to the colder waters of the Northern Hemisphere. They keep close to the edge of the ice-cap [17], [3]. There is no record of such environmental conditions on the Portuguese coast (at least since the last major glaciations ended about 10,000 years ago). This geological time scale is certainly not compatible to the presence of modern man equipped with sharp iron tools. As a consequence, bowhead whale must also be eliminated from our short list. The only possibility remaining is the North Atlantic right whale *Eubalaena glacialis*. To check out this diagnosis we did search the literature for the skeletal features of this species and there is a good match to parts PP.02.475 (Fig. 5), PP.02.486 (Fig. 6), and PP.02.509 (Fig. 7) in our sample. This comparison includes Fig. 3 in Plate IV of the classic work by Graells [10], and the relevant osteographic representations of the Balaenidae illustrated in van Beneden & Gervais [16], and in Flower [15] Comparative studies by Turner [18] and Cumbaa [19] may also be called upon to further substantiate our conclusion.

Osteological studies preferably shall include comparative analysis of specimens in reference collections. In Portugal there are no right whale skeletons available for study in the Natural History Museums. We turned instead to NOAA and Smithsonian staff and, after digital images were sent for examination, we obtained the confirmation of North Atlantic right whale diagnosis by James G. Mead, Curator Emeritus at the Smithsonian Institution (USA).

### Historical framework

**Figure 11:**
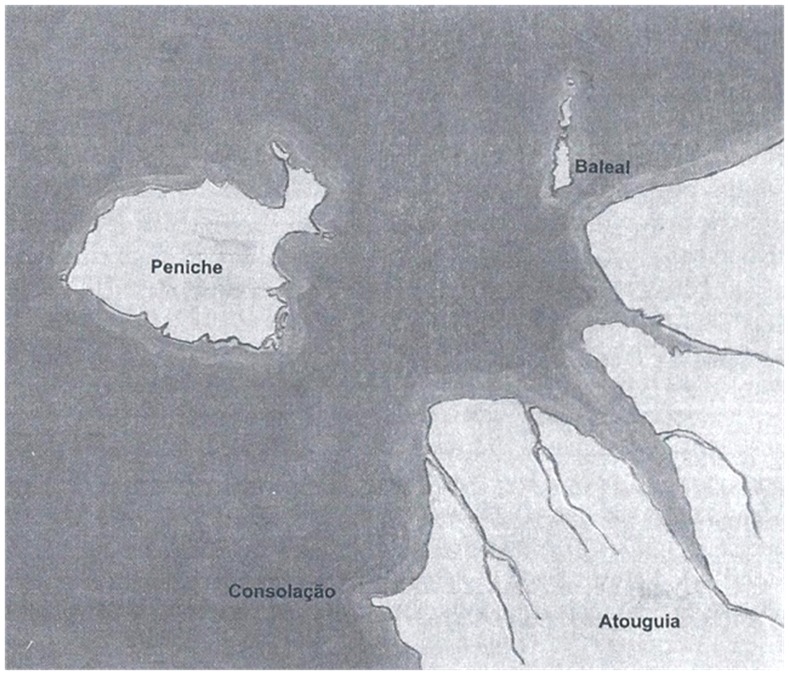
Sketch of the coastline in the 12th century (*ca*. 1147). Atouguia was a trading harbour sheltered in the mouth of a small river. Peniche was still an island offshore [22]

Interpretation of the skeletal remains of large whales found needs an historical perspective and some considerations about important changes in the contour of the shoreline there. Peniche was a rock island several hundred metres from shore and with no permanent human occupation when the Roman Empire pushed its way into the region in 61 BC [20]. The old village of Touguia (later renamed Atouguia) was an important harbour on the mainland and took part in the classic routes of sea trade reaching the coasts of Western Iberia. It was conveniently placed halfway between the Douro and Tejo rivers [21]. Peniche was still a rocky island in the 12th century (Fig. 11) and presumably stood some 1 200 m from shore [22]. A Crusader's fleet with 200 sails moored there overnight on the 27th June 1147, while travelling south to assist the first Portuguese king (Afonso Henriques) in a victorious assault to conquer Lisbon. Following military success, King Afonso Henriques gave property rights to leaders of the helping Crusaders and granted them privilege in the new conquered territories. Guillaume de La Corni (or Descornes) became landlord of *Herdade da Atouguia* in 1148 and social order was organised with two Royal decrees (*forais*) - one for the newcomers (*franci*) and another for the pre-existing population (*galeci*). These *franci* (or francs) did come from European Atlantic territories around Normandy, Flanders, and perhaps from regions in the southern coast of England. The term *galeci* applied to members of the community already established in the territory under previous Islamic rule. This population may have included elements from NW Iberia. The original arrangements made for the two groups were laid down in writing by King Sancho I. His royal decree (*foral*) was signed in 4 March 1168 and sets rights and norms that should be obeyed by members of each group. The *franci* got the uppermost social status but the *galeci* had important rights too. Many among them did work at coastal fisheries or in agriculture [21]. The *foral* of King Sancho I to the *galeci* of Atouguia in 1168 also established duties and taxes that should be paid on the produce of agriculture, cattle raising, and fishing. However, there is no reference to any taxes due on the capture or transaction of whales.

A few decades later, the “customary practice” (*costumes*) of Atouguia had confirmation in writing by the next Portuguese king, Afonso II. His document from the 4th March 1206 contains a detailed description of taxes that should be paid on the transaction of goods that were important in the local economy. Further to the tax values for trading slaves (*mouros*) and cattle, this list also includes the trade of whale products (meat and fat). This commerce was set apart from ordinary fish. Whale was much prized then and its importance relative to other merchandise may be realized from the high tax values in Table 3.

**Table 3:** The customary value of taxes due when trading selected merchandise in medieval Atouguia.

Merchandise	Quantity	Tax due
Slave (male) – *mouro*	1	1 *maravedi*
Slave (female) – *moura*	1	3 *maravedi*
Horse (male)	1	1 *maravedi*
Horse (female)	1	3 *maravedi*
Donkey (male)	1	1 *soldo*
Donkey (female)	1	6 *dinheiros*
Dried fish	1 horse load	3 *soldos*+9 *dinheiros*
Dried fish	1 donkey load	(halved)
Fresh fish	1 horse load	1 *maravedi*
Fresh fish	1 donkey load	6 *dinheiros*
Fat whale - *baleia grossa*	1 horse load	3 *maravedi*+20 *dinheiros*
Fat whale - *baleia grossa*	1 donkey load	(halved)
Thin whale - *baleia magra*	1 horse load	1 *maravedi*
Thin whale - *baleia magra*	1 donkey load	(halved)

This was established by King Afonso II in a written document from 04 March 1206 [21]. Weight of goods was set in ‘horse loads’ and in ‘donkey loads’ (one ‘horse load’ was worth two ‘donkey loads’). The words *maravedi*, *soldo* and *dinheiro* designate old currency units (listed here in decreasing order of their relative value). It is a distressing thought nowadays to realise that one horse load of “fat whale” could be worth much more than human life.

The origins of whaling activities in the area remain controversial. Scavenging of dead whales probably had been carried out for millennia. Sperm whale ivory was used in the manufacture of buttons and other objects found in archaeological sites from the Portuguese Chalcolithic [23]. There is literary evidence suggesting that whales were subject of specialized hunting during the Roman period, with specific equipment and well established procedures [24]. Opianus in *Halieutica* (II century BC) compares whale hunting to the classic military strategies of besieging a city [25]. However, the documented capture of large whales in nearshore habitats of the Atlantic coast in SW Europe was pioneered by the Basques around the 11th century [3], [26].

Atouguia was a flourishing medieval seaport and economic activities there peaked in the early 14th century, under the rule of King Dinis I. Population growth combined with intense development of agriculture and tree logging for shipbuilding. This brought more pressure upon natural resources and increased loads of sediment went into drainage basins. This led to higher rates of coastal sedimentation in some areas. The shallow bay at Atouguia silted up fast and direct access to the sea was eventually lost. All marine activities were transferred to nearby Peniche. Meanwhile the former island had become a peninsula, captured by a sand cord expanding seaward. Peniche already had fishing activities and sea trade in the early 16th century, when King Manuel I issued a new royal decree to Atouguia. His *Foral Novo* in the 1st June 1510 has a list of economic activities that should be taxed upon. Marine trade and fishing (for marine and freshwater species) are included, but there is no specific mention to whaling or any whale products. Atouguia was producing in agriculture and retained administrative coordination over this region, but there were human settlements already installed at Peniche. In the following decades Atouguia became landlocked and lost population, while Peniche did rise to become an important population centre associated to marine trade and fisheries. Eventually Peniche would obtain his own *foral* from King Filipe I of Portugal (Felipe II of Spain) and broke off from Atouguia in 1609.

**Figure 12:**
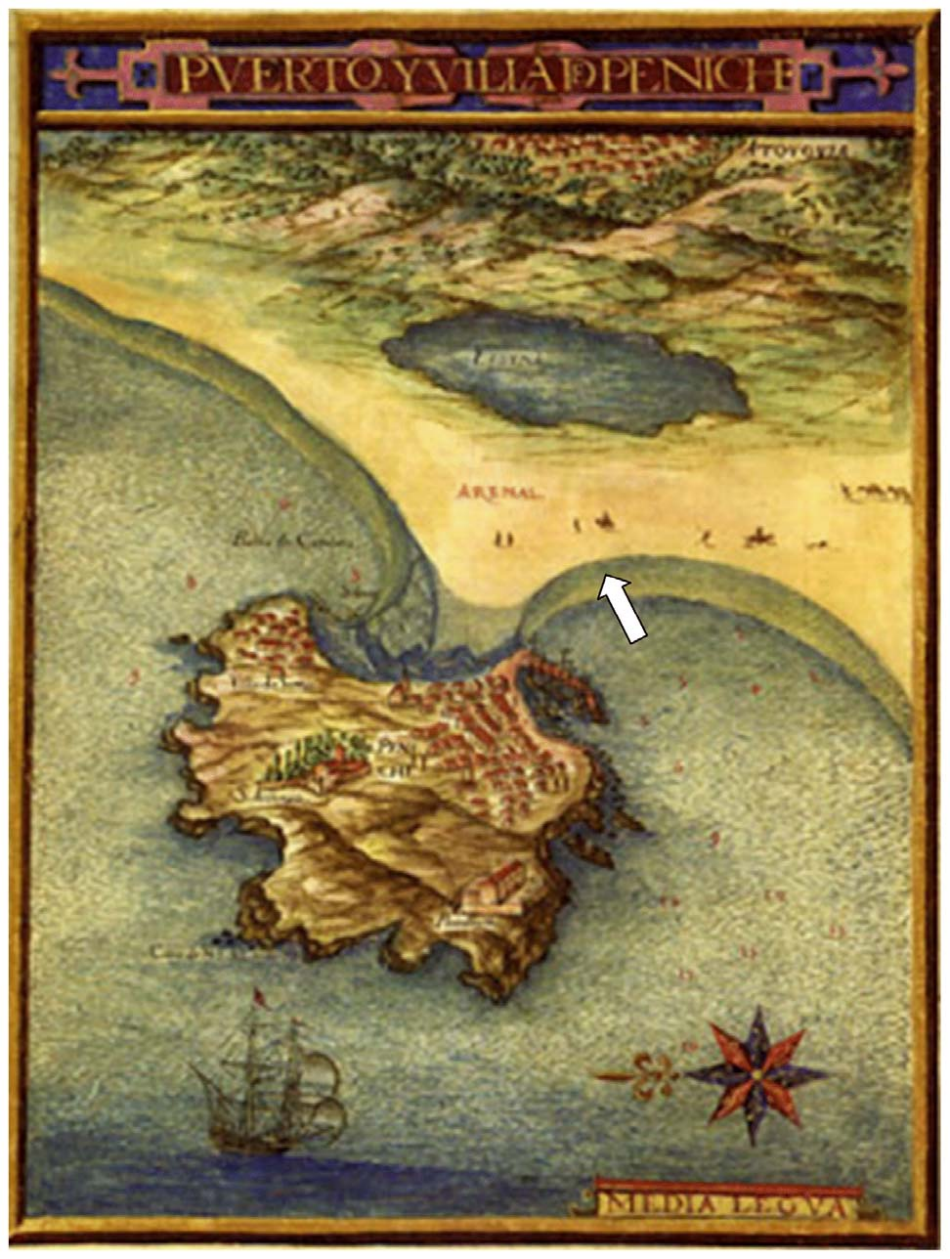
Peniche in the early 17th century, as drawn by Portuguese cosmographer Pedro Teixeira Albernaz. This map is part of an Atlas ordered by King Felipe IV of Spain and was published in 1634 (based on field surveys made in 1622–1629). The former island had been captured by a sandbar pushing the contour of the shore seaward. There was yet no road built on the dune and access on foot remained intermittent. Occasionally it was cut off by weather and tide. The position of human settlements was sketched in great detail. The harbour stands to the right in the picture, adjacent to a treacherous cut in the sandbar. The old seaport of Atouguia is now landlocked and was pictured in the background, among agricultural fields behind a coastal lagoon.

Worried about the possibility of English seaborne attacks after the Spanish Armada was defeated in 1588, King Felipe IV of Spain (Filipe III of Portugal) ordered a map survey to show details of the coastline around Iberia and a description of relevant human settlements there. An Atlas was published by Portuguese cosmographer Pedro Teixeira Albernaz in 1634 (based on field surveys from 1622 to 1629). It contains one map of Peniche that shows were the seafront was in the early 17th century (Fig. 12). Consolidation of the sandbar was in progress but no permanent terrestrial access had yet been established. There was no paved road over the sand dune leading to Peniche. Geological processes are quite dynamic in this area and they brought important changes to the local topography (Fig. 1). The whale bones described in this paper were recovered from an area of intense sand deposition. This is the place where the former island has joined the continent, when Peniche became a peninsula.

## Discussion

Whale bones were excavated at Peniche in 2001–2002 and the archaeological context suggests their dating around the late 16th to early 17th centuries. In the absence of radiocarbon dating or any other absolute methods, this timeline must be taken with caution. Nevertheless, our conclusions are fully supported by the historical information available for this area, including the position of the old seashore relative to human occupation centres. Atouguia was the old seaport while Peniche was an island offshore up to the 15th century. Economic activities at Peniche developed fast in the 16th century after the Atouguia seaport silted up.

The whale bones show evidence of human interaction, with straight cuts in different positions on the bone surface. These were probably made by expert craftsmen holding hand tools with a sharp blade. The scars are mostly in bones of the posterior occipital and in the neck area, the thoracic vertebrae, and forelimb. This further suggests some expertise in the work process. Many cuts are comparatively short but reach deep below the bone surface, suggesting vertical blows with a large instrument (axe or flensing tool). There are also thin cuts that do not penetrate deep below the surface of the bone. These were probably caused by detailed carving with hand-held knives, may be to extract meat and fat. There are also straight cuts that go all the way through on the edge of a few bones. These were obviously caused by sharp powerful blows, aimed to severe that part off from the skeleton of the whale.

No whaling instruments could be found at the worksite in 2001–2002. However, there is circumstantial evidence to suggest that the activity was going on at Peniche around the 16th to 17th centuries. The bones of large whales were found close to the lower part of the road structure. Perhaps they were used to help consolidate its foundations. The bones may have been discarded on the beach, exposed to inclement weather and winter storms. These bones are rather heavy and cumbersome. They would not have been carried for long distances over soft sand, when the road structure was not there yet to allow easy transport of large payloads on wheeled charts. Terrestrial access to Peniche in the early 17th century was restricted to walking over sand, either on foot or riding horseback. The peculiar shape of the shoreline in this area and the proximity to the seaport would be good incentive to whaling activities. Whales sighted in coastal waters could eventually be harassed at sea with small boats, trying to drive them into shallow water around the narrow pass between Peniche and the sandbar protruding from shore. Any whales trapped there would be close to their butchering site, and conveniently close to the seaport. This may lead to a wider discussion about the origins and specificity of whaling in that area.

### Origins and early development of Iberian whaling

It has been widely accepted that from its origins in the French Basque country, old whaling operations spread westwards along the northern shores of the Iberian Peninsula and were adopted by other fishing communities in coastal settlements [27]. Up to 47 ports with sustained whaling activity at some time were identified in a review by Aguilar [5] and five different countries or regions in the area were involved: French Basque country (1059), Spanish Basque country (1150), Santander (1190), Asturias (1232) and Galicia (1371). The dates refer to the first extant document giving notice of the fishery and may be taken as a rough guide to the spread of the activity in the coastal areas around the Bay of Biscay. These dates may also be compared to the information available for some areas further south on the Portuguese coast.

Whaling had great economic importance in the Middle Age and is mentioned in old documents. This stands out when we compare the value of taxes paid at Atouguia in the early 13th century (Table 3). Several Portuguese fishing communities were involved in coastal whaling [26]. A total of 38 historical sources include references to the scavenging of stranded whales or to whaling activities in mainland Portugal (1201 to 1728). There is a peak of information concerning the 13th and 14th centuries. Up to 92% of the Portuguese sources about coastal whaling are contemporary to equivalent information available for the French and Spanish Basque countries [26]. Whaling in Portugal has occurred at least since the 13th century [28], [26] and different types of whale products were introduced in the economies of several Portuguese fishing villages [29], [22]. The activity was certainly widespread on the Portuguese coast before the 14th century, which is the initial period mentioned for Galicia by Aguilar [5]. There is no evidence for temporal continuity along the coast as would be expected if a transfer of techniques had occurred from the Bay of Biscay and Galicia to the north of Portugal [26]. This may suggest that the activity in Portugal originated outside direct Basque influence.

Important political changes did occur in Iberia in the 12th and 13th centuries. These were associated to the military defeat of Muslim rule and the influx of western European crusaders to the new conquered Portuguese territories. There was intense sea trade with coastal areas in Flanders, northern France and the south coast of England. Fishing on the Portuguese coast was also widespread and the medieval populations there certainly used marine resources available to them [29]. Eventually they incorporated previous influences from the Roman period and the classic Mediterranean cultures [24], [30]. Portuguese references about whaling are rather inconclusive about techniques and the species captured, but it should be inferred that only very simple technology was used. This probably included lookouts, open boats travelling close to shore, and hand thrown harpoons [25]. There is one historical source [29] about the “black whale” hunted at Pederneira, which was a small seaport some 20 nautical miles (*ca.* 37 km) to the north of Atouguia. The name certainly fits the North Atlantic right whale and it was also mentioned by Aguilar [4].

### Zooarchaeology of the whale remains

Considering the typology of the whale bones excavated, it is worth pointing out that the sample, now preserved in the local museum, contains no less than 38 pieces with a comparatively large size. Most of these bones were in or around the skull area of large whale specimens. Other skeletal parts were left *in situ* at the worksite because they were trapped in concrete and could not be removed to the museum. These were mostly large items and they further distort our sample towards the prevalence of large bones. They compare favourably to the 42 bone fragments with rather small size recovered in the area. Some of these fragments had also been part of large bones before they got broken down. Such a prevalence of large whale bones in a comparatively small area on the old seafront is worth considering in detail.

We have skeletal remains of no less than three specimens of the North Atlantic right whale *E. glacialis*. All of them had large body size and many of their bones show obvious signs of human interaction that may be associated to whaling activity. The bones were found close to the old village and shipping areas in Peniche. This may be considered another case of the ‘schlepp effect’. Given the incipient technology available to deal with the large size and heavy weight of dead whales brought ashore, we may assume that whale carcasses were butchered on the beach. The larger bones were probably discarded *in situ*, while the meat, fat, oil and baleen would have been removed in small parcels and consumed (or traded) elsewhere. It should be noted that all transportation had to be carried out walking over soft sand in a dune habitat. This certainly was good incentive to leave behind any whale parts of considerable weight and size, provided they had little commercial value.

Also, we could find no jawbones or any identifiable fragments of them at the worksite. This is especially noteworthy because there should have been a minimum of six such bones (in line with the three sets of neck vertebrae). Jawbones are the largest bones in the whale skeleton and we can only speculate on the causes for their absence. Of course the easiest answer is that they were somewhere around the worksite and we just missed them. Nevertheless, we have found three sets of fused neck vertebrae and a number of much smaller bones from the skull (not to mention minute pottery fragments). This suggests that other possible justifications shall be considered. We know that jawbones of the large whales were taken eventually for their oil and a variety of different uses. There is a profusion of nice examples from the British Isles and other areas illustrated by Redman [31] but most of them relate to comparatively recent whaling episodes (19th and early 20th centuries). These bones were often shipped over long distances, from the capture sites to their final destination ashore in farms and towns. Often they were used as fences in cottages or rubbing posts for cattle, but also for decorative purposes.

In fact we ignore what did happen to the jawbones of the whales excavated at Peniche in 2001–2002. We have no evidence to support that they were eventually removed from the beach, but this possibility shall not be overlooked. We must recall that in nearby Atouguia da Baleia there is the jawbone of a large Balaenopterid on public display inside the S. Leonardo church [26], which some decades ago was in an old farm at Peniche. It had been there for a long time, as part of the structure of an old stone wall. There was also a local tradition of using whale bones for many different purposes. This included incorporating odd whale vertebrae as building blocks in masonry works, or taking these to use them as convenient chopping anvils.

### Historical occurrence of right whales in Portugal

It is generally accepted that the North Atlantic right whale *E. glacialis* was the main prey of coastal whaling in the Middle Age and in the pre-modern period. We have no evidence yet to ascertain the historical period in which the North Atlantic right whale ceased to be a regular visitor to Portuguese waters. We must recall that the foral of King Afonso II in 1206 addresses whales and trade of whale products at Atouguia, while the new foral of King Manuel I in 1510 does not. The reason for this remains unclear but probably it has little to do with the population dynamics of the whales. Instead we may be tempted to speculate that whaling and trade in whale products was a flourishing activity in the 13th and 14th centuries (associated to the Flanders and Hanseatic sea routes) but later it had lost popularity among the Portuguese seamen. In the 15th and early 16th centuries Portugal was picking up the riches of recently established oceanic routes and overseas trade with Africa, India, Southeast Asia and Brazil. Whaling in home waters probably was not so much attractive anymore. The situation may have changed again in the late 16th and in the first half of the 17th century. Previous access to the overseas trade routes was disrupted in relation to a crisis in Royal succession, followed by temporary loss of national independence and six decades of political union to the Crown of Spain (1580–1640). Whaling at Peniche in this period would be in line with an historical peak of the regional catches reported for Galicia and Asturias, which was estimated at around 1601–1650 by Du Pasquier [32].

Anyway, a sharp decline in the number of right whales in Iberian waters must have occurred from the 17th to the 18th century. These dates are in line with decreases reported by Aguilar [5] for the Bay of Biscay. They also agree with equivalent reports by Markham [33] and Fischer [34] mentioned in Duguy [35]. In the mid 19th century Bocage [36] makes no reference to *E. glacialis* in his short list of cetaceans (and pinnipeds) observed in Portugal. Assessment of catch statistics in the 20th century also shows that no right whales were reported by the industrial whaling fleet land-based at Setúbal [37]. This Norwegian-style Shore whaling [38] was active in mainland Portugal for two short periods after both World War conflicts. Modern methods were used to help finding targets and to kill the whales. The absence of right whale records must be emphasised because the actual number of large cetaceans taken by that industry was quite important on a regional scale (587 whales were killed in 1925–1927, and 584 whales in 1944–1951). The main prey was the fin whale *Balaenoptera physalus*. They were hunted mostly on the edge of the continental shelf, between Cape da Roca and Cape Sines. Later on, an inventory by Teixeira [39] has no stranding records of right whales on the Portuguese Iberian coast. None come out either in a recent review by Sousa & Brito [40].

Seven right whale kills were reported in the Azores between 1873 and 1888, but the last reliable whaling records in the NE Atlantic were obtained in Madeira, with captures still in the 20th century [37]. Maul & Sergeant [41] reported a mother and calf taken on 27 February 1967 just off the northern coast of Madeira island. These were killed by the whaling industry based at Caniçal. A third specimen (adult) was also reported, but eventually it was able to evade capture. The European population of *E. glacialis* is now presumed extinct [42], [43]. Very few recent sightings of this species have been reported in the Eastern Atlantic and these probably result from stray movements of individuals from the NW Atlantic population [7]. A review by Silva *et al.* [44] describes the sighting of one right whale of Western Atlantic origin less than one mile to the south of Pico island (in the Azores) on 5 January 2009. Photographs were obtained and a match was found to right whale no. 3270 in the NARW Catalogue. This whale had been previously sighted on 24 September 2008, on the Bay of Fundy, Canada.

Northern Atlantic right whales now remain the most critically endangered from all the large whales [1], and human action was decisive to reach this condition. Nevertheless, we prefer to leave the positive note of one observation reported two decades ago from Galicia [45], and a mother with calf sighted from shore at Cape St. Vincent, off SW. Portugal [46]. Research on the former occurrence and biology of right whales *E. glacialis* on the Portuguese coast is urgently needed and reliable information about this species in the area is still fragmentary. We must understand the historical distribution and patterns of extinction of this species on a regional basis, as it may provide significant contributions to the conservation of extant populations of right whales worldwide.
